# Elucidating the function of STING in systemic lupus erythematosus through the STING Goldenticket mouse mutant

**DOI:** 10.1038/s41598-024-64495-6

**Published:** 2024-06-17

**Authors:** Pichpisith Pierre Vejvisithsakul, Chisanu Thumarat, Asada Leelahavanichkul, Nattiya Hirankan, Trairak Pisitkun, Soren Riis Paludan, Prapaporn Pisitkun

**Affiliations:** 1 Program in Translational Medicine, Faculty of Medicine Ramathibodi Hospital, Mahidol University, Bangkok, Thailand; 2 Division of Allergy, Immunology, and Rheumatology, Department of Medicine, Faculty of Medicine Ramathibodi Hospital, Mahidol University, Bangkok, Thailand; 3 Department of Microbiology, Faculty of Medicine, Chulalongkorn University, Bangkok, Thailand; 4 Translational Research in Inflammation and Immunology Research Unit (TRIRU), Department of Microbiology, Chulalongkorn University, Bangkok, Thailand; 5 Centre of Excellent in Immunology and Immune-Mediated Diseases, Department of Microbiology, Faculty of Medicine, Chulalongkorn University, Bangkok, Thailand; 6 Center of Excellence in Systems Biology, Faculty of Medicine, Chulalongkorn University, Bangkok, Thailand; 7 Department of Biomedicine, Aarhus University, Aarhus, Denmark

**Keywords:** STING, Tmem173, Pristane-induced lupus, Mutation, SLE, Lupus nephritis, Autoimmunity

## Abstract

The complexity of systemic lupus erythematosus (SLE) arises from intricate genetic and environmental interactions, with STING playing a pivotal role. This study aims to comprehend the function of STING using the pristane-induced lupus (PIL) model in *Sting* missense mutant mice (*Goldenticket* or *Sting*^*Gt*^), which contrasts with previous research using *Sting* knockout mice. Investigating two-month-old *Sting*^*Gt*^ mice over six months post-PIL induction, we observed a profound reduction in autoimmune markers, including antinuclear and anti-dsDNA antibodies, germinal center B cells, and plasma cells, compared to their wild-type counterparts. A pivotal finding was the marked decrease in IL-17-producing T cells. Notably, the severity of lupus nephritis and pulmonary hemorrhages was significantly diminished in *Sting*^*Gt*^ mice. These findings demonstrate that different genetic approaches to interfere with STING signaling can lead to contrasting outcomes in SLE pathogenesis, which highlights the need for a nuanced understanding of the role of STING in drug development for SLE. In summary, the loss of *Sting* function in *Goldenticket* mutant mice rescued autoimmune phenotypes in PIL. This study reveals the critical nature of STING in SLE, suggesting that the method of STING modulation significantly influences disease phenotypes and should be a key consideration in developing targeted therapies.

## Introduction

Type I interferon (IFN) is one of the critical cytokines that induce lupus disease and is essential for Pristane-induced lupus (PIL)^1^. The interruption of type I IFN receptor ameliorates lupus-like phenotypes in NZB (New Zealand Black) mice^2^. The mice with *Tlr7* (Toll-like receptor 7) over-expression developed lupus phenotypes^3,4^, while the *Tlr7*-deficient mice did not develop glomerulonephritis in PIL^5^. In addition, the *Tlr9* (Toll-like receptor 9) -deficient C57BL/6 mice develop less histological renal injury and immune complex deposition than wild-type (WT) mice in PIL^6^. However, PIL in *Tlr9*-deficient BALB/C mice shows worsening renal pathology compared to WT^7^. These data suggest that interfering with the upstream of type I IFN production through nucleic acid sensors in different backgrounds showed different outcomes of lupus diseases.

TREX1 (three prime repair exonuclease 1) degrades excess intracellular single-strand DNA to prevent the activation of interferon-stimulated genes^8^. *Trex1*-deficient mice develop autoimmunity mediated through type I IFN signaling^9^. STING (stimulator of interferon genes) or Tmem173 (Transmembrane protein 173) is a cytosolic DNA sensor that responds to dsDNA or cyclic GMP-AMP (cGAMP). The cGAS (Cyclic GMP–AMP synthase) processes dsDNA and releases cGAMP^10^. The absence of cGAS and Sting signaling abrogates the lethal autoimmune phenotypes in *Trex1*-deficient mice^11,12^. In addition, gain-of-function STING mutations have been identified in patients with monogenic autoinflammatory disease and type I interferonopathies, so-called STING-associated vasculopathy with onset in infancy (SAVI)^13–17^, inflammatory lupus-like disease and familial chilblain lupus^18,19^.

The deletion of *Sting* aggravates inflammation and increases autoantibody production in the autoimmune MRL*.Fas *^*lpr*^ mice^20^. PIL shows the autoimmunity development in the *Sting* knockout mice, which depends on DNA^21^. A mutant mouse strain, *Goldenticket* (*Gt*), that harbors a point mutation (T596A) in *Sting* results in an isoleucine-to-asparagine substitution (I199N) in the Sting protein which leads to the loss of STING function and type I IFN signaling^22^. Activation of STING signaling induces B cell death, and the *Sting*^*Gt*^ mutant mice develop accelerated autoimmune arthritis in collagen-induced arthritis (CIA)^23^. Nevertheless, STING signaling in dendritic cells (DC) promotes plasmacytoid DC differentiation and initiates lupus phenotypes in the 129/B6.*Fcgr2b* (Fc gamma receptor 2b) -deficient mice^24^. The STING inhibitor (ISD017) targeted STING signaling specifically and affected lupus development in the lupus-prone 129/B6.*Fcgr2b*-deficient mice^25^. These data suggested that STING is a gatekeeper of autoimmunity in specific contexts.

The STING is an endoplasmic reticulum adaptor that has several domains, each with specific roles and interactions, including transmembrane proteins (TM), dimerization domain (DD), Cyclic-di-GMP-binding domain (CBD), and C-terminal tail (CTT)^26,27^. These domains interact with adaptor proteins that enhance inflammatory response or inhibit type I IFN production^28,29^. The C-terminal region of STING is both necessary and sufficient to activate TBK1 and stimulate the phosphorylation of IRF3^30^.

The cGAS-STING activating canonical pathway can lead to TBK1/IRF3 mediated type I IFN expression. Several functions of non-canonical signaling downstream of STING have been described, such as autophagy, cellular condensation, and DNA damage repair^10^. The genetic manipulation of *Sting* in different domains (mutant or deletion) could lead to opposite outcomes in the same mouse model.

Although the *Sting*-deletion mice developed PIL^20^, the *Sting*^*Gt*^ mutant prevented the *Fcgr2b*-deficient mice from developing lupus disease^24^. Thus, we aim to verify whether a different genetic manipulation of *Sting* could lead to different outcomes by introducing PIL into the *Sting*^*Gt*^ mutant mice. We found that PIL in the *Sting*^*Gt*^ mice did not develop anti-dsDNA and lupus nephritis. Also, the *Sting*^*Gt*^ mice limited the expansion of plasmacytoid dendritic cells after pristane injection and reduced IFN-gamma and IL-17-producing double-negative T cells. Our data suggested that STING plays a vital role in SLE pathogenesis, and inhibition of STING at the specific sequence could lead to a potential target for therapeutic intervention.

## Methods

### Pristane-induced lupus mouse model

The *Sting*^*Gt*^ mice (the Goldenticket or *Tmem173*^*Gt*^) mice were created via chemically inducing mutagen with N-ethyl-N-nitrosourea (ENU)^22^. The C57BL/6 WT and *Sting*^*Gt*^ mice (8–10 weeks old) were intraperitoneally injected with 500 µl of Pristane or tetramethylpentadecane (TMPD) (#P2870, SIGMA-ALDRICH Co., MO, USA)^31^. Blood samples were collected before and after 6 months after pristane injection intraperitoneally. The mice were monitored for clinical symptoms and humanely sacrificed if they showed distressing symptoms. Mice were bred and housed at the Faculty of Medicine, Chulalongkorn University. All experiments were performed with the approval of the Animal Experimentation Ethics Committee of Chulalongkorn University Medical School with all relevant institutional guidelines (007/2561). All methods were performed following the ARRIVE guidelines.

### Sample collection and preparation

The organs were harvested from the mice six months after pristane injection. The splenocytes were isolated, and cold ACK lysis buffer was added to lyze red blood cells for 5 min. The splenocytes were washed and resuspended in 0.5% BSA in PBS for flow cytometry. Kidneys were fixed with 4% Paraformaldehyde in PBS. Lungs were perfused with normal buffer formalin to inflate the lungs and fixed with 4% Paraformaldehyde in PBS for tissue pathology.

### Detection of anti-dsDNA and antinuclear antibody (ANA)

The quantitative ELISA for anti-dsDNA was performed from the sera collected six months after pristane injection using the previous protocol^24^. The ANA was tested in the sera (dilution 1:2000) of the mice using Hep-2 cells, following the described protocol^24^. The researcher was blinded to the treatment groups and graded the fluorescence intensity as 4 = maximal fluorescence (brilliant yellow-green), 3 = less brilliant (yellow-green fluorescence), 2 = definite (dull yellow-green), and 1 = very dim (subdued fluorescence)^19^.

### Flow cytometry analysis

Splenocytes were resuspended with staining buffer (0.5%BSA in PBS and 0.09% azide) to obtain the 20 × 10^6^ cells/ml concentration. The splenocytes (1 × 10^6^ cells) were stained with flow antibody including CD4 (GK1. 5; #100423), CD8 (53–6. 7; #100708), CD62L (MEL-14; #104417), CD44 (IM7; #103035), CD3ε (145-2C11; #100312), ICOS (C398.4A; #313517), CD11c (N418; #117312), B220 (RA3-6B2; #103222), CD11b (M1/70; #101228), I-Ab (AF6-120.1; #116406), PDCA-1 (129c1; #127103), CD80 (16-10A1; #104733), GL7 (GL7; #144604), CD138 (281–2; #142506), F480 (BM8; #123112), CD45RB (C363-16A; #103307), Ly6c (HK1.4; #128022), Ly6g (1A8; #127608), IgM (RMM-1; #406512), IgD (11-26c.2a; #405718), CD21 (7E9; #123415), CD23 (B3B4; #101613), CD19 (6D5; #115,522), IFNγ (XMG1.2; #505821), and IL17A (TC11-18H10.1; #506921), (Biolegend, San Diego, CA, USA), FAS (15A7; #12095181) (Invitrogen, Frederick, MD, USA). Cell viability (eBioscience™ Fixable Viability Dye eFluor™ 780, #65-0865-14, Thermo Fisher Scientific, CA, USA). For cell surface marker staining, splenocytes were isolated and prepared as previously described^32^.

### Intracellular staining for cytokine-induced cells

The splenocytes were resuspended with staining buffer (0.5%BSA in PBS and 0.09% azide) to obtain the 20 × 10^6^ cells/ml concentration. The 200-µL cells were then seeded, which is equivalent to 1 × 10^6^ cells, in a culture medium with 25 ng/ml PMA, 1 ug/ml ionomycin (Sigma-Aldrich, Darmstadt, Germany), and 1X GolgiPlug (brefeldin A, Biolegend, San Diego, CA, USA) into each well of the 96-well culture plate. The cells were incubated at 37˚C, 5% CO_2_ for 4 h. Afterward, cells were stained with surface markers, i.e., anti-CD3, anti-CD4, and anti-CD8 antibodies, and fixed in 200 µl of fixation buffer (BioLegend, San Diego, CA, USA) at 4 °C overnight without light exposure. Cells were centrifuged and subjected to permeabilization to stain intracellular cytokine using 50 µL of 1X permeabilization buffer (BioLegend, San Diego, CA, USA). The cells were stained with anti-IFNγ and anti-IL17A antibodies. The flow cytometry was performed within 2 h after intracellular staining.

### Immunohistochemistry

The mice were euthanized 6 months after the pristane injection. The whole lung was infused with paraformaldehyde to dilate the alveoli through the trachea and then immersed in 4% paraformaldehyde/PBS. The harvested kidneys and lungs were fixed with 4% paraformaldehyde/PBS and embedded in paraffin. The 5 µm of tissue sections were stained with Hematoxylin and eosin (H&E). The kidney scores were graded as glomerular and interstitial scores previously described^30^. In brief, glomerular scores were defined as 0 = normal; 1 = focal, mild, or early proliferative; 2 = moderate or definite proliferative; 3 = diffuse and focal or diffuse proliferative; 4 = severe diffuse proliferative with crescent/sclerosis, and interstitial scores were defined as 0 = normal; 1 = focal or small pockets (10–15 cells) of mononuclear cells (MNC); 2 = focal infiltrates (15–30 cells); 3 = multifocal extensive infiltrates with necrosis; 4 = multifocal or diffuse and extensive with necrosis. The lung pathology was evaluated as diffuse pulmonary hemorrhage and interstitial inflammation^31,32^. In short, interstitial inflammatory infiltrates were graded on a scale of 0–3 (none, mild, moderate, severe), and the percentage of lungs with hemorrhage was estimated and assigned the following scores: 0, no hemorrhage; 1, 0–25%; 3, 25–50%; 3, 50–75%; and 4, 75–100%.

### Serum creatinine

We detected the creatinine level from the mouse sera using the QuantiChromTM Creatinine Assay Kit (Cat. No. DICT-500, BioAssay Systems, Hayward, CA, USA). The standard creatinine was diluted to a concentration of 2 mg/dL according to the manufacturer's protocol for blood samples. 30 µL of serum from each sample was duplicated into a 96-well plate. Then, prepare the working reagent by mixing 100 µL each of reagents A and B, quickly adding 200 µL of working reagent into each well, and briefly tap to mix. The absorbance (OD) was read out immediately as OD_0_ and at 5 min as OD_5_ at 510 nm. The ODs were calculated using the equation below to obtain the serum creatinine concentration.

$$Serum\,\, creatinine \,\,concentration=\frac{{OD}_{SAMPLE\,\,5}-{OD}_{SAMPLE\,\,0}}{{OD}_{STD\,\,5}-{OD}_{STD\,\,0}}\times \left[\text{STD}\right] mg/dL.$$

### Statistical analysis

The descriptive statistics of the study characteristics were expressed as mean ± standard deviation (SD) for continuous variables and frequencies (percentages) for categorical variables. To compare two independent sample means from the same population where the data did not meet the assumptions of normality, the Mann–Whitney *U*-test, a non-parametric statistical test, was employed. A two-way Analysis of Variance (ANOVA) was utilized for the analysis, which involved multiple comparisons across different groups. Subsequent post hoc analyses were conducted using Tukey's multiple comparisons test to explore specific differences between the wild-type (WT) and *Sting*^*Gt*^ genotypes and between the control and pristane-induced lupus (PIL) treatment groups. The statistical analyses were performed using GraphPad Prism software (version 7, San Diego, CA, USA). The results for all experiments are presented as mean ± standard deviation (SD). Statistical significance was established at p values < 0.05, with significance levels: *p ≤ 0.05, **p ≤ 0.01, and ***p < 0.001.

## Results

### STING mediated autoantibody production in pristane-induced lupus

**Figure 1:**
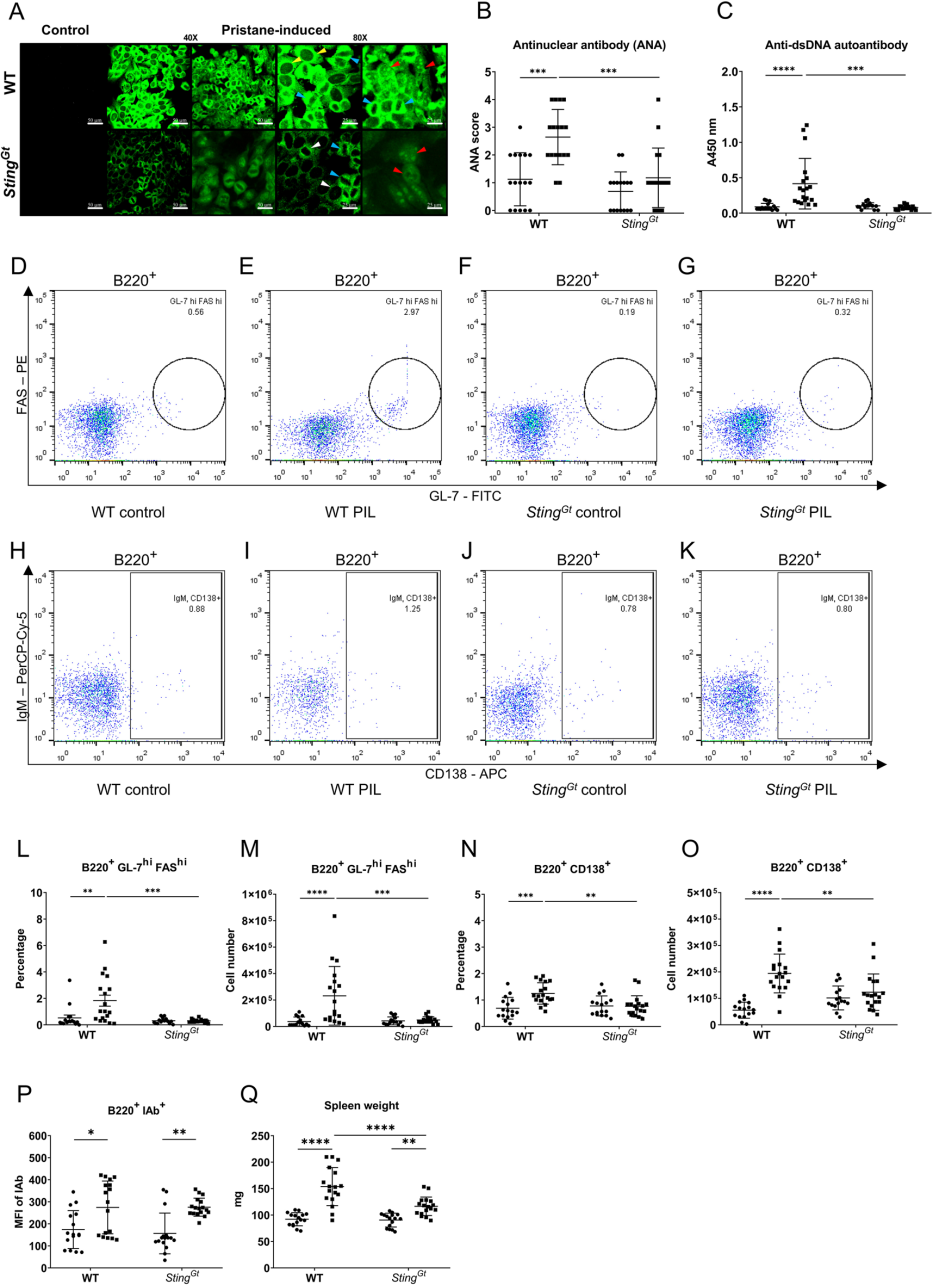
STING mediated autoantibody production in pristane-induced lupus. (**A**) Confocal microscopy shows staining of an antinuclear antibody (ANA) of WT (*Sting*^*wt/wt*^) and *Sting*-deficient (*Sting*^*Gt*^) mouse sera at 1:2000 dilution. The ANA patterns show cytoplasmic homogenous (yellow arrow), mitotic (blue arrow), rim (white arrow), and nucleolar (red arrow). Scale bar = 50 μm at 40X magnification and 25 μm at 80X magnification. (**B**) The fluorescence intensity of ANA from the serum of the WT and *Sting*^*Gt*^ mice was scored at the dilution 1:2000. (**C**) Detection of anti-double-stranded DNA (dsDNA) (1:100) using ELISA, read out at the wavelength of 450 nm. (**D**–**K**) Flow cytometry analysis of splenocytes shows gating strategy of (**D**–**G**) B220^+^GL7^hi^FAS^hi^ (germinal center B cells) and (**H**–**K**) B220^+^CD138^+^ (Plasma cells) from WT and *Sting*^*Gt*^ mice. The splenocytes of WT and *Sting*^*Gt*^ mice show the percentage of (**L**) germinal center B cells (B220^+^GL-7^hi^FAS^hi^) and (**N**) plasma cells (B220^+^CD138^+^) out of B cells, and cell number of (**M**) germinal center B cells and (**O**) plasma cells. (**P**) The mean fluorescence intensity (MFI) of the IAb expression in the B220^+^ cell population. (**Q**) Spleen weight of WT and *Sting*^*Gt*^ mice were measured. Cell numbers are shown per spleen. Data are shown as mean ± SD;* p*-values were indicated as *p < 0.05, **p < 0.01, ***p < 0.001. Dot ● indicates control, and square ■ indicates PIL (N = 16–18 mice/group).

Autoreactive B cells produced the antinuclear antibody (ANA), the autoantibody commonly found in lupus disease. Pristane induces the production of autoantibodies, including those targeting anti-Sm, RNP, dsDNA, chromatin, and ribosomal P^33,34^. We tested for ANA from the sera collected six months after pristane injection to demonstrate whether STING affects autoantibody production. The pattern of ANA showed similarity in both C57/BL6-WT and *Sting*^*Gt*^ mice. However, the intensity of ANA staining identified by immunofluorescence was significantly reduced in *Sting*^*Gt*^ mice compared to C57/BL6-WT mice (Fig. 1A,B). Also, the level of anti-dsDNA titers was not increased by pristane injection in the *Sting*^*Gt*^ mice (Fig. 1C).

*Sting/Tmem173* is involved in spontaneous germinal center B cell development and plasma cell differentiation, requiring autoantibody production in *Fcgr2b*-deficient mice^24^. The germinal center B cells were characterized by B220^+^GL-7^hi^FAS^hi^ (Fig. 1D–G), and plasma cells were identified by B220^+^CD138^+^ (Fig. 1H–K). The expansion of germinal center B cells (Fig. 1L,M) and plasma cells (Fig. 1N,O) was absent in the *Sting*^*Gt*^ mice, whereas the WT mice exhibited an increase following pristane injection. However, Pristane increased the mean fluorescence intensity of MHC (IAb) on B cells, representing the antigen-presenting ability in both WT and *Sting*^*Gt*^ mice B cells (Fig. 1P). Furthermore, the spleen weight increased in WT and *Sting*^*Gt*^ mice after PIL. However, spleens from *Sting*^*Gt*^ mice were significantly smaller than those from WT mice (Fig. 1Q). We also observed that PIL-injected WT mice exhibited a smaller size and consumed less food than their PIL-injected *Sting*^*Gt*^ counterparts. The data suggest that *Sting*^*Gt*^ mice exhibited reduced levels of anti-dsDNA, decreased germinal center formation, and limited plasma cell expansion after pristane injection.

### STING facilitated the activation of innate immunity in pristane-induced lupus

**Figure 2:**
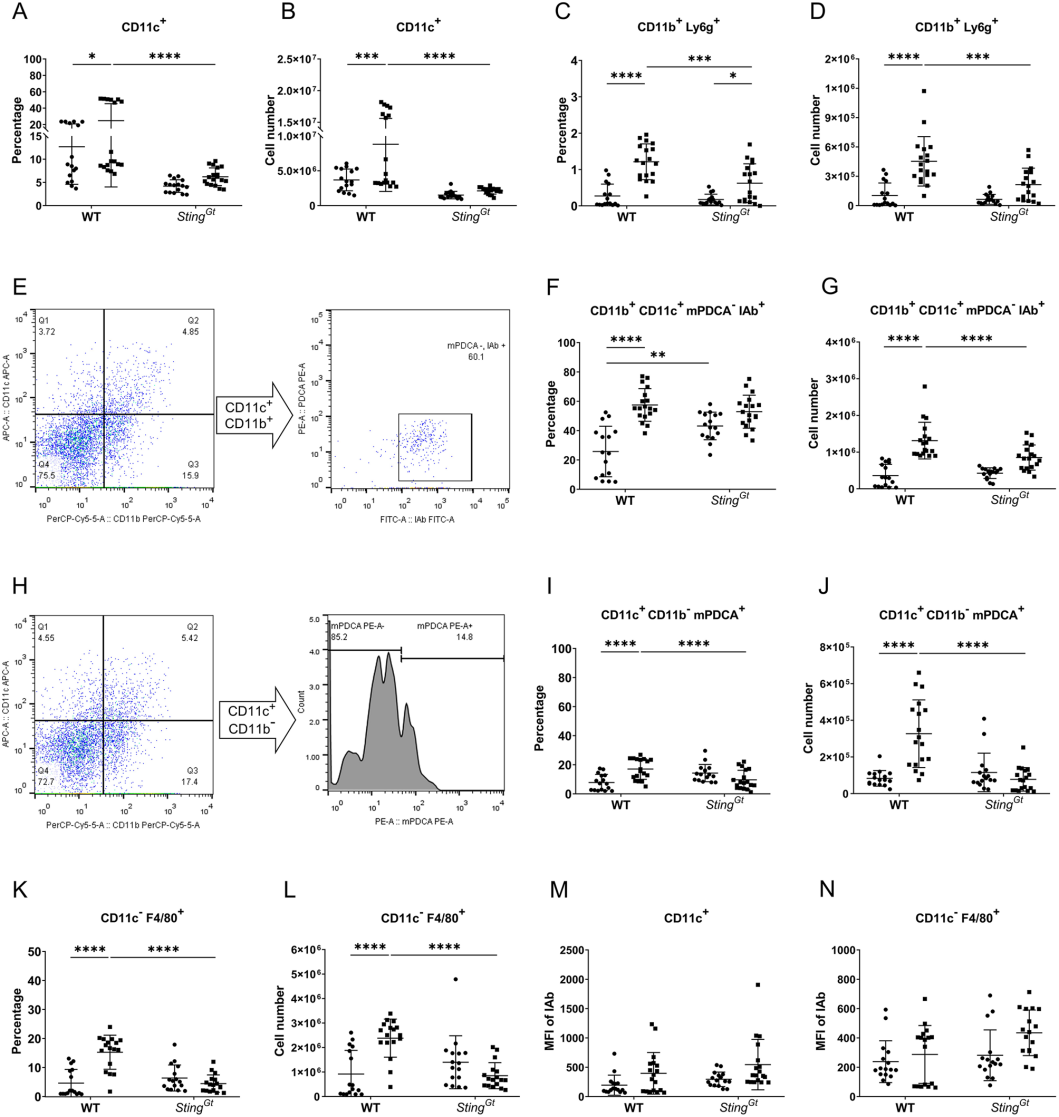
STING facilitated plasmacytoid dendritic cells and macrophage expansion in pristane-induced lupus. The isolated splenocytes from WT and *Sting*^*Gt*^ mice were stained and analyzed by flow cytometry. Data show the proportion and the cell number of (**A**,**B**) DC (CD11c^+^), (**C**,**D**) CD11b^+^ Ly6g^+^ cells (neutrophils) out of splenocytes. The gating strategy of (**E**) CD11b^+^ CD11c^+^ mPDCA^-^ IAb^+^ (conventional dendritic cells or cDCs) population and (**H**) CD11c^+^ CD11b^-^ mPDCA^+^ (plasmacytoid dendritic cells or pDCs). The proportion and cell number of the (**F**,**G**) cDCs out of CD11c^+^CD11b^+^), (**I**,**J**) pDCs out of CD11c^+^CD11b^-^, and (**K**,**L**) CD11c^-^ F4/80^+^ (macrophages) out of splenocytes. (**M**,**N**) The mean fluorescence intensity (MFI) of the IAb expression in the (M) CD11c^+^ cell and (**N**) CD11c^-^ F4/80^+^ population. Cell numbers are shown per spleen. Data are shown as mean ± SD; *p*-values were indicated *p < 0.05, **p < 0.01, ***p < 0.001, and ****p < 0.0001. Dot ● indicates control, and square ■ indicates PIL (N = 16–18 mice/group).

Dendritic cell (DC) expansion did not occur in *Sting*^*Gt*^ mice following pristane injection (Figs. 2A,B). While the percentages of neutrophils increased in WT and *Sting*^*Gt*^ mice after pristane injection (Fig. 2C), the absolute number of neutrophils in PIL-treated *Sting*^*Gt*^ mice was significantly lower than in WT mice (Fig. 2D). Classical DCs (cDC) were gated based on CD11c^+^CD11b^+^IAb^+^mPDCA^-^ markers (Fig. 2E). While WT mice exhibited an expansion of classical DCs following PIL, *Sting*^*Gt*^ mice did not show this increase (Fig. 2F,G). Next, we analyzed plasmacytoid dendritic cells (pDCs) based on gating with CD11c^+^CD11b^-^mPDCA^+^ markers (Fig. 2H). Once again, WT mice increased pDCs following PIL, while *Sting*^*Gt*^ mice did not exhibit this change (Fig. 2I,J). Additionally, pristane increased macrophage (CD11c^-^F480^+^) expansion in WT mice but not in *Sting*^*Gt*^ mice (Fig. 2I,J). However, neither WT nor *Sting*^*Gt*^ mice showed a rise in MHC expression in CD11c^+^ cells and macrophages after pristane injection (Fig. 2K,L). The data suggested STING participated in cDCs, pDCs, and macrophage expansion in pristane-induced lupus.

### STING is involved in double-negative T-cell expansion and IL-17A production

**Figure 3:**
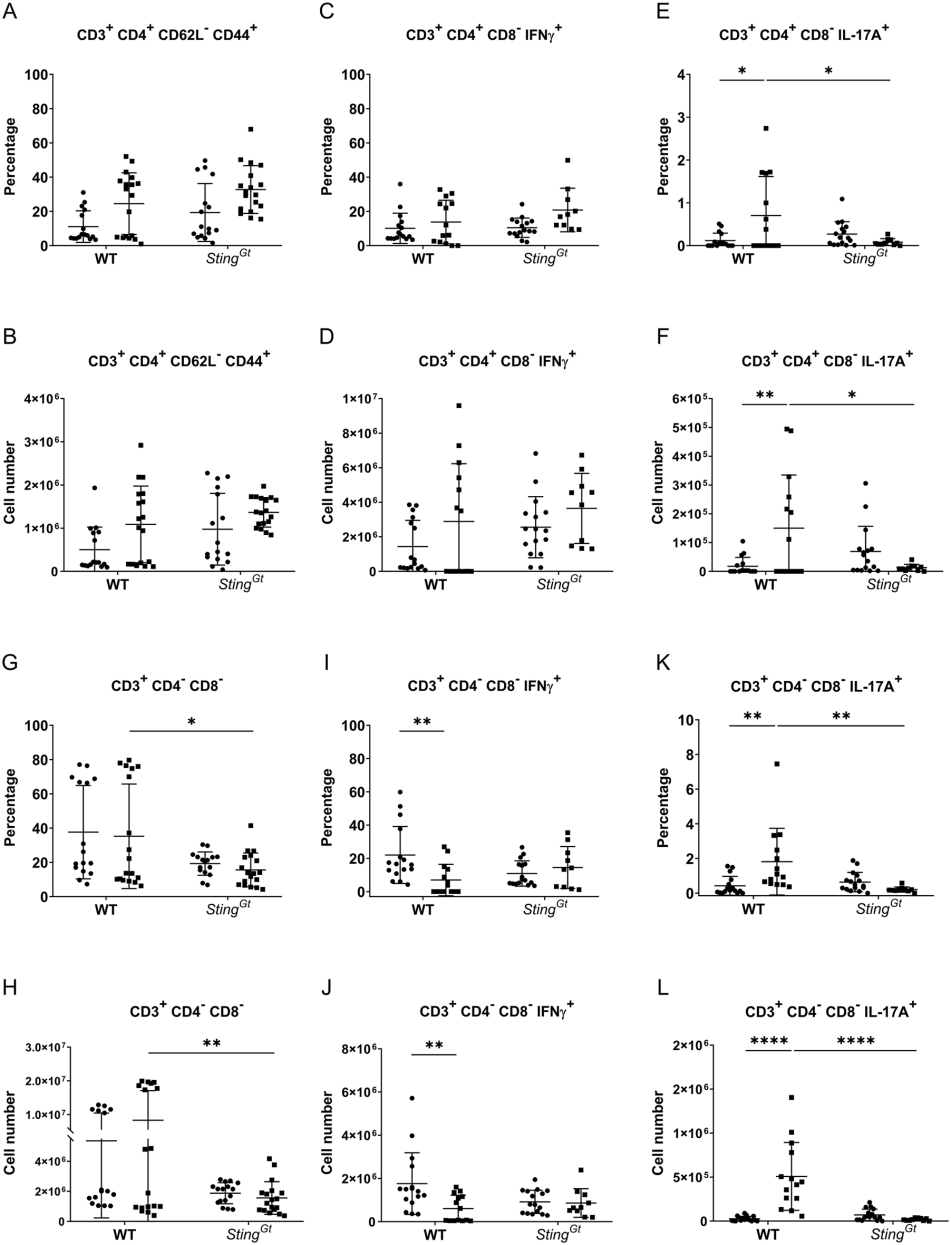
STING involved in double-negative T cell expansion and IL-17A production. The isolated splenocytes from WT and *Sting*^*Gt*^ mice were stained and analyzed by flow cytometry. Data show the proportion and the absolute number of cells (**A**,**B**) effector memory T cells (CD3^+^CD4^+^CD62L^-^CD44^+^) out of CD4^+^ T cells, (**C**,**D**) IFN-γ producing T helper cells (CD3^+^ CD4^+^CD8^-^IFNγ^+^) out of CD4^+^ T cells, (**E**,**F**) IL-17A producing T helper cells (CD3^+^CD4^+^CD8^-^IL-17A^+^) out of CD4^+^ T cells, (**G**,**H**) double-negative T cells (CD3^+^CD4^-^CD8^-^) out of CD3^+^ T cells, and (**I**,**J**) IFN-γ producing double-negative T cells (CD3^+^CD4^-^CD8^-^IFNγ^+^) out of double-negative T cells, and (**K**,**L**) IL-17A producing double-negative T cells (CD3^+^CD4^-^CD8^-^IL-17A^+^) out of double-negative T cells. Cell numbers are shown per spleen. Data are shown as mean ± SD; *p*-values were indicated *p < 0.05, **p < 0.01, ***p < 0.001, and ****p < 0.0001. Dot ● indicates control, and square ■ indicates PIL (N = 10–18 mice/group).

Activated DC primed naive T cells to become effector memory cells. The expansion of effector memory T cells in the *Fcgr2b*-deficient mice declined without STING signaling^24^. However, the populations of effector memory T cells (Fig. 3A,B) and IFN-γ-producing T helper cells (Fig. 3C,D) did not increase in WT and *Sting*^*Gt*^ mice following PIL. While pristane induced IL-17A-producing T helper cells in WT mice, this effect was not observed in *Sting*^*Gt*^ mice (Fig. 3E,F). We examined DN-T cells in PIL-treated mice and did not observe an increase in this population in either WT or *Sting*^*Gt*^ mice (Fig. 3G,H). However, a comparison between PIL-treated mice revealed a significantly lower abundance of DN-T cells in *Sting*^*Gt*^ mice than in WT mice (Fig. 3G,H). Double-negative (DN) T cells are known to produce both IFN-γ and IL-17A in *Fcgr2b*-deficient lupus mice^35^. The STING antagonist has been shown to reduce double-negative (DN) T cells in Fcgr2b-deficient mice^25^. Subsequently, we analyzed cytokine-producing DN T cells following PIL and observed a reduction specifically in IL-17A^+^ DN-T cells (Fig. 3K,L) rather than IFN- γ^+^ DN-T cells (Fig. 3I,J) in *Sting*^*Gt*^ mice. The data suggest that STING participates in IL-17A production from both T helper and DN T cells.

### STING facilitates glomerulonephritis in pristane-induced lupus

**Figure 4:**
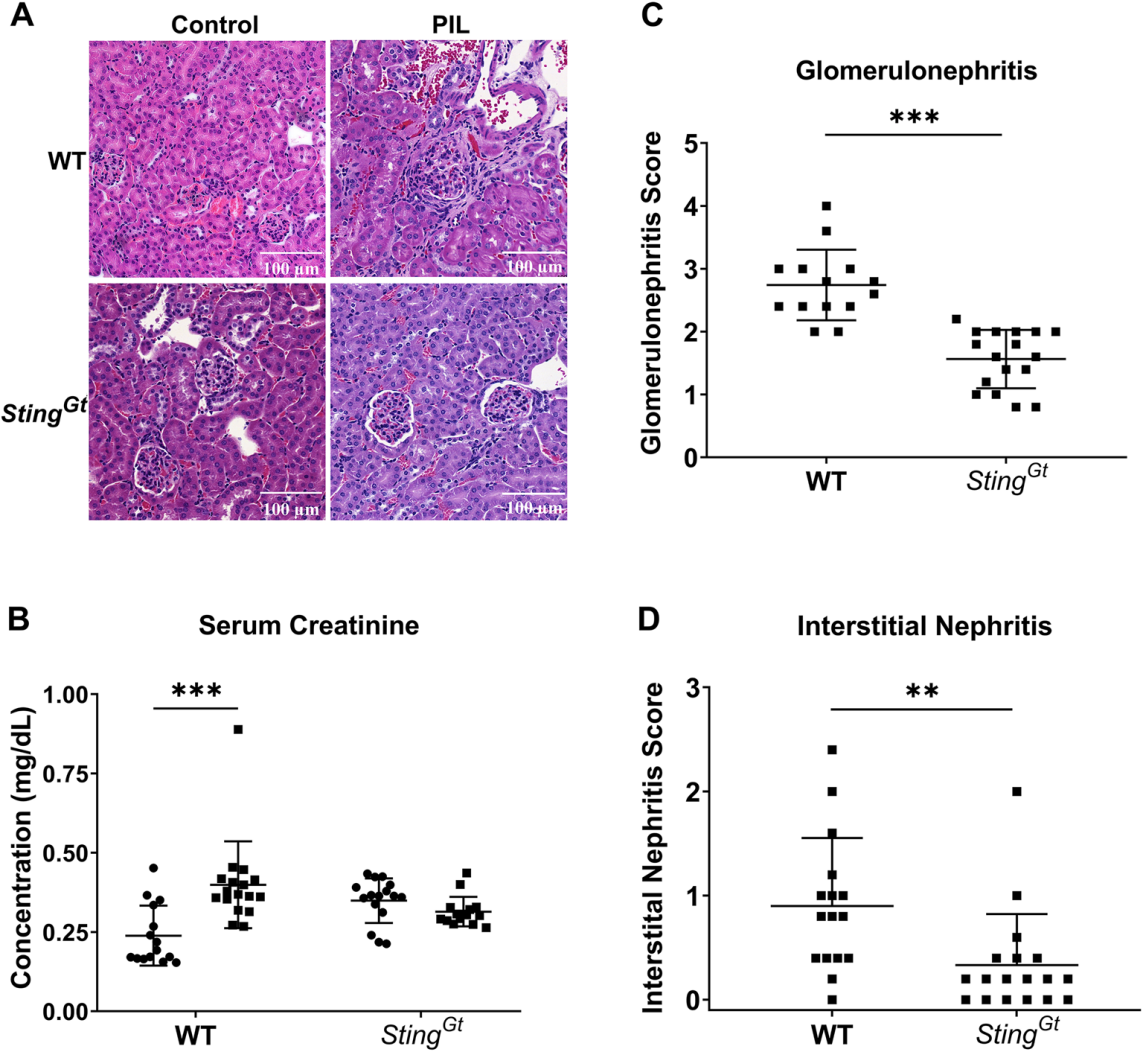
STING facilitates glomerulonephritis in pristane-induced lupus. The tissues (kidney and lung) were sectioned and stained with Hematoxylin and eosin stain (H&E). (**A**) The section of kidneys from pristane-induced mice showed glomerulonephritis (scale bar 100 µm). (**B**) Serum creatinine (mg/dL). The kidney histopathology was graded for (**C**) glomerulonephritis (glomerular score) and (**D**) interstitial nephritis (interstitial score). Data are shown as mean ± SD. *p*-values were indicated *p < 0.05, **p < 0.01, and ***p < 0.001. (N = 16–18 mice/group).

The kidney sections were stained using Hematoxylin and Eosin (H&E). Microscopic examinations revealed that WT and *Sting*^*Gt*^ mice exhibited increased cellularity and mesangial expansion in the glomeruli (Fig. 4A). However, a notable difference was observed in the WT mice, which presented more severity with fibrocellular crescents (Fig. 4A). While WT mice exhibited increased serum creatinine following PIL, *Sting*^*Gt*^ mice maintained stable serum creatinine levels (Fig. 4B). Furthermore, the *Sting*^*Gt*^ mice demonstrated lower glomerular and interstitial scores upon pristane injection than the WT mice (Fig. 4C,D). These findings suggest that *Sting*^*Gt*^ mice were more resistant to develop chronic glomerulonephritis.

### STING mediates enhanced pulmonary hemorrhage in response to pristane-induced lupus

**Figure 5:**
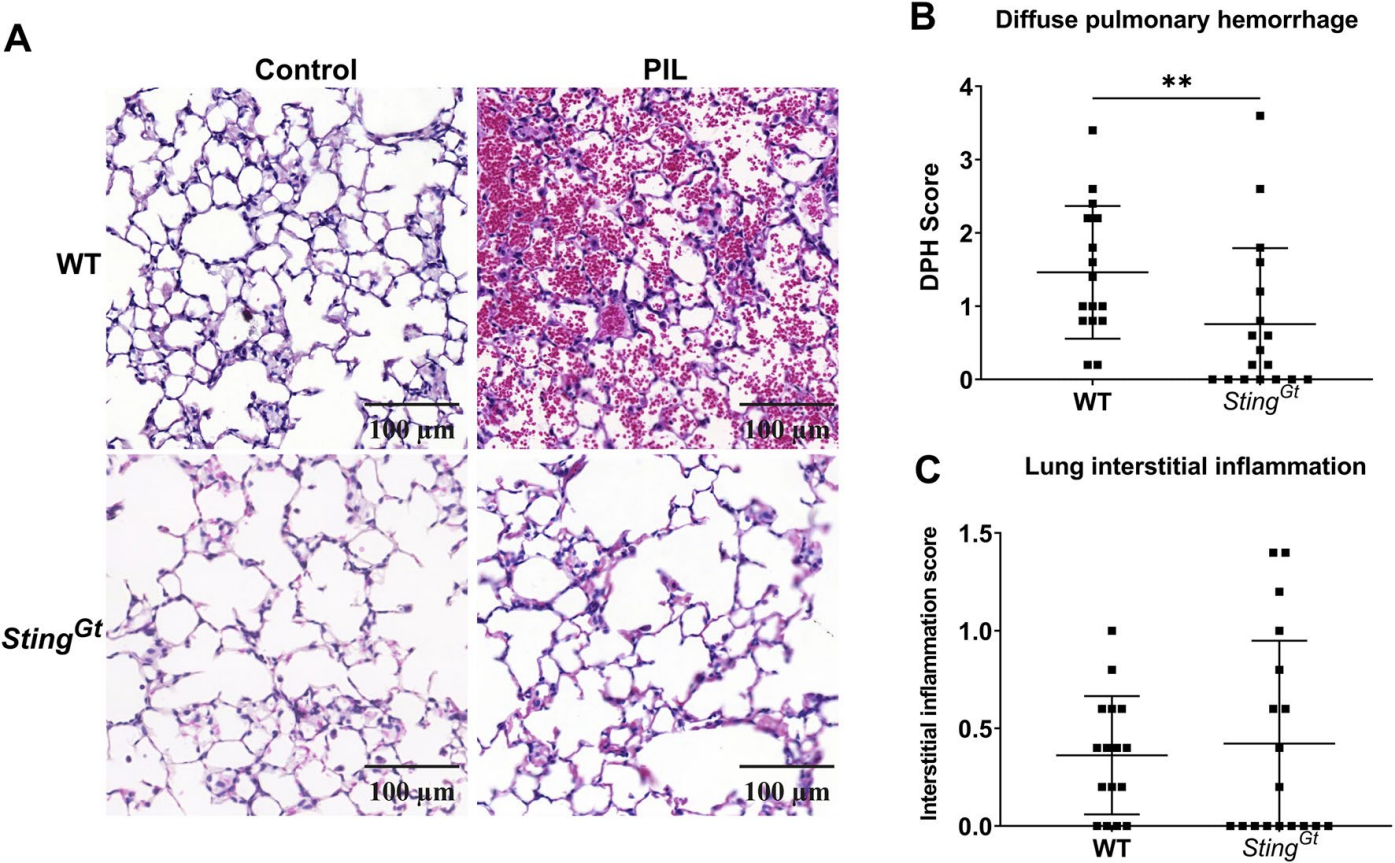
STING mediates enhanced pulmonary hemorrhage in response to pristane-induced lupus. (**A**) The lung sections showed diffuse pulmonary hemorrhage from pristane-induced mice (scale bar 100 µm). (**B**,**C**) The lung histopathology was graded for (**B**) diffuse pulmonary hemorrhage and (**C**) interstitial inflammation scores. Data are shown as mean ± SD. *p*-values were indicated *p < 0.05, **p < 0.01, ***p < 0.001. (N = 16–18 mice/group).

Our study revealed that PIL notably induced more severe pulmonary hemorrhage in WT mice than interstitial infiltration (Fig. 5A). In contrast, lungs from *Sting*^*Gt*^ mice exhibited a significantly reduced inflammatory response compared to their WT counterparts (Fig. 5A). Pathological examination highlighted PIL-induced diffuse pulmonary hemorrhage in WT mice, which was markedly less pronounced in *Sting*^*Gt*^ mice (Fig. 5A,B). Interestingly, PIL did not significantly affect interstitial inflammation scores between WT and *Sting*^*Gt*^ mice (Fig. 5C). These findings suggest a pivotal role of STING in mediating pulmonary hemorrhage in response to PIL.

## Discussion

The diversity in lupus mouse models points to distinct mechanisms driving disease progression^31^—for instance, the MRL*.Fas *^*lpr*^ mice lack interferon (IFN) signatures but rely on STING-mediated signaling. Interestingly, the disease exacerbates when STING is absent^20,31^. On the other hand, *Trex1*-deficient mice, which require type I IFN-mediated signaling, benefit from the lack of STING signaling, showing improved conditions^9,11,12^. The 129/B6.*Fcgr2b*-deficient mice, carrying autoimmune susceptibility genes from the 129 loci, exhibit an increased IFN signature but can survive without the STING-mediated pathway^24^. This variation suggests that the role of STING in disease development may be highly model-specific, with the presence of a type I IFN signature in lupus mice potentially indicating the significance of STING in specific models.

Our study showed a decrease of antinuclear antibodies (ANA), anti-dsDNA, germinal center B cells, and plasma cells in the *Sting* mutant or *Goldenticket* (*Sting*^*Gt*^) mice, which are findings that align with those reported in the 129/B6.*Fcgr2b*-deficient mice^24^. However, the observed cytoplasmic staining pattern on Hep-2 cells in the Gt mice may indicate the persistence of other antigen specificities. Additionally, the *Sting*^*Gt*^ mice exhibited a decrease in the expansion of pDCs, cDCs, neutrophils, and macrophages following pristane injection, highlighting the STING-dependent nature of pDC differentiation^24^. Interestingly, despite these reductions, the mean fluorescence intensity of IAb on CD11c^+^ cells and macrophages was not different between WT and *Sting*^*Gt*^ mice following PIL. This suggests that PIL did not increase the MHC-II expression in the macrophages.

In 129/B6.*Fcgr2b*-deficient mice, activated conventional dendritic cells (cDC) were found to promote T effector memory cell (Tem) differentiation^24^. While in 129/B6.*Fcgr2b*-deficient mice, the absence of STING reduced Tem expansion; the pristane-induced lupus model similarly elicited Tem induction in both *Sting*^*Gt*^ and WT mice. This discrepancy in Tem phenotypes between the two models may be linked to activated cDC's ability to prime T cells effectively. Lupus mouse models and studies in human Systemic Lupus Erythematosus (SLE) have demonstrated an increase in double-negative T cells (DN-T) capable of producing both IFN-γ and IL-17A^35–38^. Inhibition of the IL-17 signaling pathway has been shown to alleviate glomerulonephritis^35,39^. Notably, following pristane injection in *Sting*^*Gt*^ mice, there was a reduction in IL-17A-producing T cells but not IFN-γ^+^ T cells. The decrease of IL-17A may contribute to the reduced severity of lupus nephritis observed.

In this study, PIL led to lupus-related symptoms, including glomerulonephritis and diffuse pulmonary hemorrhage, in WT mice. This allowed for a comparative analysis of Sting loss-of-function in Goldenticket mutant mice (*Sting*^*Gt*^) concerning lupus progression. The severity of lupus nephritis and diffuse pulmonary hemorrhage in *Sting*^*Gt*^ mice was significantly reduced compared to WT mice following pristane injection, indicating that *Sting*^*Gt*^ was involved in PIL pathogenesis. In contrast, a prior study using *Sting* knockout mice revealed increased antinuclear antibodies, peritoneal macrophages, and CD11b^+^ Ly6C^hi^ inflammatory monocytes in the spleen. However, Pristane failed to induce lupus nephritis in their WT counterparts^21^. Sting deletion accelerates autoimmunity and inflammatory monocytes through endosomal Toll-like receptors^21^. Additionally, mice deficient in *cGAS* developed PIL through non-canonical inflammasome activation in macrophages^40^ and were reliant on DNA-mediated signaling^21^. These findings highlight multiple redundant pathways of DNA activating nucleic acid sensors in PIL pathogenesis.

The discrepancies observed in a previous study and this study could be attributed to different genetic disruption approaches of STING. In *Sting*-deficient mice, targeting different genetic regions can influence functionality, necessitating a specific STING sequence for signaling or interaction with downstream molecules. *Sting* knockout mice in a previous study were created by removing exons 2 to 5, which include the cyclic dinucleotide binding (CBD) domain^27^. Conversely, NLRC3 inhibits STING trafficking and its association with TBK1 by interacting with STING's ligand-binding domain (amino acids 139-344) and NLRC3's nucleotide-binding domain^28^. This loss may weaken the NLRC3's inhibitory interaction. The *Goldenticket* mutant mice (*Sting*^*Gt*^) were generated by the N-Ethyl-N-Nitrosourea-induced mutagenesis feature a mutation (T596A) leading to a change in the amino acid I199N^22^, which located in the CBD domain^40^. While STING GT (null) and STING S365A mice do not induce type I IFN, only STING S365A mice retain intact autophagy^41^. The single nucleotide mutation in *Sting*^*Gt*^ mice is critical for the type I interferon response to c-di-GMP and c-di-AMP^22^ but may not affect NLRC3 binding.

Their genetic backgrounds may influence the variability in disease severity observed in lupus models. Models exhibiting high type I interferon (IFN) activity have demonstrated a dependency on the STING signaling pathway^24,42^, whereas MRL. *Fas*^*lpr*^ mice, influenced by endosomal Toll-like receptors (TLRs), display contrasting results^20,43^. Furthermore, a previous study showed that pristane injection did not induce typical lupus phenotypes, such as glomerulonephritis and diffuse pulmonary hemorrhage^21^, possibly indicating a lower expression of type I IFN in that context and impacting STING signaling dynamics. Consequently, it remains challenging to conclusively determine whether disrupting STING signaling is an appropriate therapeutic target for SLE treatment.

The intricate nature of lupus mouse models reflects the varied mechanisms underlying systemic lupus erythematosus (SLE). Mutations in the STING gene have been linked to human autoinflammatory diseases and interferonopathies^15,18^. While SLE patients may exhibit abnormalities in type I IFN signaling, it is essential to note that not all conditions characterized by interferonopathies manifest as SLE. Notably, the targeted STING inhibitor ISD017 has shown effectiveness in reducing glomerulonephritis in *Fcgr2b*-deficient mice, as well as diminishing ISG responses in peripheral blood mononuclear cells (PBMC) from SLE patients^25^. This evidence underscores the potential role of intrinsic STING function in the pathogenesis of human SLE.

Our research indicates that targeting STING-mediated signaling could be a promising intervention approach. While specific mouse models displayed lackluster outcomes in STING inhibition for lupus treatment, these variations could stem from the genetic backgrounds of the mice and the intrinsic processes driving type I IFN production. Given the intricate interplay between STING and other molecular adaptors, strategies to inhibit STING in systemic lupus erythematosus (SLE) treatment must be meticulously crafted to optimize efficacy and minimize adverse effects.

## Data Availability

All relevant data have been presented in the manuscript. Requests for or questions about the data can be addressed to Prapaporn.pis@mahidol.ac.th.

## References

[1] Nacionales DC, Kelly-Scumpia KM, Lee PY, Weinstein JS, Lyons R, Sobel E (2007). Deficiency of the type I interferon receptor protects mice from experimental lupus. Arthritis Rheum..

[2] Santiago-Raber ML, Baccala R, Haraldsson KM, Choubey D, Stewart TA, Kono DH (2003). Type-I interferon receptor deficiency reduces lupus-like disease in NZB mice. J. Exp. Med..

[3] Pisitkun P, Deane JA, Difilippantonio MJ, Tarasenko T, Satterthwaite AB, Bolland S (2006). Autoreactive B cell responses to RNA-related antigens due to TLR7 gene duplication. Science.

[4] Deane JA, Pisitkun P, Barrett RS, Feigenbaum L, Town T, Ward JM (2007). Control of toll-like receptor 7 expression is essential to restrict autoimmunity and dendritic cell proliferation. Immunity.

[5] Savarese E, Steinberg C, Pawar RD, Reindl W, Akira S, Anders HJ (2008). Requirement of Toll-like receptor 7 for pristane-induced production of autoantibodies and development of murine lupus nephritis. Arthritis Rheum..

[6] Summers SA, Hoi A, Steinmetz OM, O'Sullivan KM, Ooi JD, Odobasic D (2010). TLR9 and TLR4 are required for the development of autoimmunity and lupus nephritis in pristane nephropathy. J. Autoimmun..

[7] Bossaller L, Christ A, Pelka K, Nundel K, Chiang PI, Pang C (2016). TLR9 deficiency leads to accelerated renal disease and myeloid lineage abnormalities in pristane-induced murine lupus. J. Immunol..

[8] Yang YG, Lindahl T, Barnes DE (2007). Trex1 exonuclease degrades ssDNA to prevent chronic checkpoint activation and autoimmune disease. Cell.

[9] Stetson DB, Ko JS, Heidmann T, Medzhitov R (2008). Trex1 prevents cell-intrinsic initiation of autoimmunity. Cell.

[10] Chen C, Xu P (2022). Cellular functions of cGAS-STING signaling. Trends Cell Biol..

[11] Gall A, Treuting P, Elkon KB, Loo YM, Gale M, Barber GN (2012). Autoimmunity initiates in nonhematopoietic cells and progresses via lymphocytes in an interferon-dependent autoimmune disease. Immunity.

[12] Gray EE, Treuting PM, Woodward JJ, Stetson DB (2015). Cutting edge: cGAS is required for lethal autoimmune disease in the Trex1-deficient mouse model of Aicardi-Goutieres syndrome. J. Immunol..

[13] Wobma H, Shin DS, Chou J, Dedeoglu F (2022). Dysregulation of the cGAS-STING pathway in monogenic autoinflammation and lupus. Front. Immunol..

[14] Demirkaya E, Sahin S, Romano M, Zhou Q, Aksentijevich I (2020). New horizons in the genetic etiology of systemic lupus erythematosus and lupus-like disease: Monogenic lupus and beyond. J. Clin. Med..

[15] Liu Y, Jesus AA, Marrero B, Yang D, Ramsey SE, Sanchez GAM (2014). Activated STING in a vascular and pulmonary syndrome. N. Engl. J. Med..

[16] d'Angelo DM, Di Filippo P, Breda L, Chiarelli F (2021). Type I interferonopathies in children: An overview. Front. Pediatr..

[17] Keskitalo S, Haapaniemi E, Einarsdottir E, Rajamaki K, Heikkila H, Ilander M (2019). Novel TMEM173 mutation and the role of disease modifying alleles. Front. Immunol..

[18] Jeremiah N, Neven B, Gentili M, Callebaut I, Maschalidi S, Stolzenberg MC (2014). Inherited STING-activating mutation underlies a familial inflammatory syndrome with lupus-like manifestations. J. Clin. Investig..

[19] Konig N, Fiehn C, Wolf C, Schuster M, Cura Costa E, Tungler V (2017). Familial chilblain lupus due to a gain-of-function mutation in STING. Ann. Rheum. Dis..

[20] Sharma S, Campbell AM, Chan J, Schattgen SA, Orlowski GM, Nayar R (2015). Suppression of systemic autoimmunity by the innate immune adaptor STING. Proc. Natl. Acad. Sci. U. S. A..

[21] Motwani M, McGowan J, Antonovitch J, Gao KM, Jiang Z, Sharma S (2021). cGAS-STING pathway does not promote autoimmunity in murine models of SLE. Front. Immunol..

[22] Sauer JD, Sotelo-Troha K, von Moltke J, Monroe KM, Rae CS, Brubaker SW (2011). The N-ethyl-N-nitrosourea-induced Goldenticket mouse mutant reveals an essential function of Sting in the in vivo interferon response to Listeria monocytogenes and cyclic dinucleotides. Infect. Immun..

[23] Tansakul M, Thim-Uam A, Saethang T, Makjaroen J, Wongprom B, Pisitkun T (2020). Deficiency of STING promotes collagen-specific antibody production and B cell survival in collagen-induced arthritis. Front. Immunol..

[24] Thim-Uam A, Prabakaran T, Tansakul M, Makjaroen J, Wongkongkathep P, Chantaravisoot N (2020). STING mediates lupus via the activation of conventional dendritic cell maturation and plasmacytoid dendritic cell differentiation. iScience.

[25] Prabakaran T, Troldborg A, Kumpunya S, Alee I, Marinkovic E, Windross SJ (2021). A STING antagonist modulating the interaction with STIM1 blocks ER-to-Golgi trafficking and inhibits lupus pathology. EBioMedicine..

[26] Wu X, Wu FH, Wang X, Wang L, Siedow JN, Zhang W (2014). Molecular evolutionary and structural analysis of the cytosolic DNA sensor cGAS and STING. Nucleic Acids Res..

[27] Ishikawa H, Barber GN (2008). STING is an endoplasmic reticulum adaptor that facilitates innate immune signalling. Nature.

[28] Zhang L, Mo J, Swanson KV, Wen H, Petrucelli A, Gregory SM (2014). NLRC3, a member of the NLR family of proteins, is a negative regulator of innate immune signaling induced by the DNA sensor STING. Immunity.

[29] Gaidt MM, Ebert TS, Chauhan D, Ramshorn K, Pinci F, Zuber S (2017). The DNA inflammasome in human myeloid cells is initiated by a STING-cell death program upstream of NLRP3. Cell.

[30] Tanaka Y, Chen ZJ (2012). STING specifies IRF3 phosphorylation by TBK1 in the cytosolic DNA signaling pathway. Sci. Signal..

[31] Zhuang H, Szeto C, Han S, Yang L, Reeves WH (2015). Animal models of interferon signature positive lupus. Front. Immunol..

[32] Kumpunya S, Thim-Uam A, Thumarat C, Leelahavanichkul A, Kalpongnukul N, Chantaravisoot N (2022). cGAS deficiency enhances inflammasome activation in macrophages and inflammatory pathology in pristane-induced lupus. Front. Immunol..

[33] Satoh M, Kumar A, Kanwar YS, Reeves WH (1995). Antinuclear antibody production and immune-complex glomerulonephritis in BALB/c mice treated with Pristane. Proc. Natl. Acad. Sci. U. S. A..

[34] Satoh M, Reeves WH (1994). Induction of lupus-associated autoantibodies in BALB/c mice by intraperitoneal injection of Pristane. J. Exp. Med..

[35] Pisitkun P, Ha HL, Wang H, Claudio E, Tivy CC, Zhou H (2012). Interleukin-17 cytokines are critical in development of fatal lupus glomerulonephritis. Immunity.

[36] Rafael-Vidal C, Perez N, Altabas I, Garcia S, Pego-Reigosa JM (2020). Blocking IL-17: A promising strategy in the treatment of systemic rheumatic diseases. Int. J. Mol. Sci..

[37] Zhang Z, Kyttaris VC, Tsokos GC (2009). The role of IL-23/IL-17 axis in lupus nephritis. J. Immunol..

[38] Crispin JC, Oukka M, Bayliss G, Cohen RA, Van Beek CA, Stillman IE (2008). Expanded double negative T cells in patients with systemic lupus erythematosus produce IL-17 and infiltrate the kidneys. J. Immunol..

[39] Lee SY, Lee SH, Seo HB, Ryu JG, Jung K, Choi JW (2019). Inhibition of IL-17 ameliorates systemic lupus erythematosus in Roquin(san/san) mice through regulating the balance of TFH cells, GC B cells, Treg and Breg. Sci. Rep..

[40] Zhang Z, Zhou H, Ouyang X, Dong Y, Sarapultsev A, Luo S (2022). Multifaceted functions of STING in human health and disease: From molecular mechanism to targeted strategy. Signal. Transduct. Target Ther..

[41] Yamashiro LH, Wilson SC, Morrison HM, Karalis V, Chung JJ, Chen KJ (2020). Interferon-independent STING signaling promotes resistance to HSV-1 in vivo. Nat. Commun..

[42] Ahn J, Gutman D, Saijo S, Barber GN (2012). STING manifests self DNA-dependent inflammatory disease. Proc. Natl. Acad. Sci. U. S. A..

[43] Christensen SR, Shupe J, Nickerson K, Kashgarian M, Flavell RA, Shlomchik MJ (2006). Toll-like receptor 7 and TLR9 dictate autoantibody specificity and have opposing inflammatory and regulatory roles in a murine model of lupus. Immunity.

